# Content and face validity of a food frequency questionnaire for the assessment of ultra-processed food consumption in the Italian adult population: a pilot study

**DOI:** 10.3389/fnut.2026.1817542

**Published:** 2026-06-05

**Authors:** Erica Cardamone, Federica Fiori, Giulia Carioni, Francesca Iacoponi, Laura Rossi, Marco Silano, Umberto Agrimi, Maria Parpinel

**Affiliations:** 1 Department of Medicine, University of Udine, Udine, Italy; 2 Unit of Human Nutrition and Health, Department of Food Safety, Nutrition and Veterinary Public Health, Italian National Institute of Health, Rome, Italy; 3 Division of Epidemiology and Biostatistics, IEO European Institute of Oncology IRCSS, Milan, Italy; 4 Department of Cardiovascular, Endocrine-Metabolic Diseases and Aging, Italian National Institute of Health, Rome, Italy

**Keywords:** content validity, dietary intake, face validity, food frequency questionnaire, ultra processed food

## Abstract

**Background:**

The multidimensional complexity of dietary intake, coupled with the current uncertainty around the Ultra-Processed Foods (UPFs) concept, underscores the need for tailored, valid, and reliable dietary assessment tools. This pilot study assessed face and content validity of the novel modified NOVA Food Frequency Questionnaire (mNOVA FFQ), designed to assess UPF consumption among Italian adults, to finalize a version for subsequent testing.

**Methods:**

A sample of 52 Italian volunteers from two institutions was recruited. Participants self-completed an initial mNOVA FFQ version, followed by an online/in-person semi-structured interview. A trained interviewer administered 12 questions evaluating understanding/interpretability of the mNOVA FFQ, and a 24 h dietary recall (24HR) to better characterize eating habits in the target population and inform potential refinements to the food list. Both quantitative and qualitative analyses were performed to evaluate the content and face validity of the FFQ.

**Results:**

Twenty FFQ items exhibited predominantly negative responses (>50%) to the main questions. All foods reported in the 24HRs were represented in the FFQ, indicating comprehensive coverage of participant diets. Thirteen FFQ items were not consumed in the 24 h prior to the interview, and four of these were consumed by less than 1% of Italian adults, according to national data. Overall, participants perceived the questionnaire as clear and generally easy to understand, although thematic analysis of their feedback highlighted several areas for improvement in the FFQ.

**Conclusion:**

This study enabled refinement of the questionnaire closed list of food items and structure, and enhance its clarity and understandability, underscoring the importance of evaluating both content and face validity, and involving the target population in the development of new tools. Further investigation is required to establish the reliability and criterion validity of the mNOVA FFQ for its application within the Italian adult population.

## 1 Introduction

Dietary intake is defined as “multidimensional (i.e., it is a complex, multi-layered exposure and behavior) and dynamic (i.e., it varies over time and life course developmental stage)” (1). People consume foods and drinks in different combinations and patterns, determining a multidimensional complexity that should be considered during the development of a dietary assessment tool (2). This complexity further increases when considering highly processed foods (HPFs), as current food processing classification systems rely on qualitative conceptual criteria, resulting in substantial ambiguity and scientific controversy (3, 4). Among these, the most widely adopted system in the literature is the NOVA classification, which is theoretically grounded in the extent and purposes of food processing and divides foods into four categories: (I) unprocessed or minimally processed foods, (II) processed culinary ingredients, (III) processed foods, and (IV) ultra-processed foods (UPFs) (5).

Despite its widespread use, one of the main methodological limitations of the available evidence on UPF consumption lies in the dietary assessment tools employed, which were not developed specifically for this purpose (6, 7). Indeed, only a limited number of studies have aimed to validate tools for assessing UPF consumption (8), highlighting the need for further research in this area.

As defined by Frongillo et al. (9) “Validation is the process of determining whether a measure or indicator is suitable for providing useful analytical measurement for a given purpose and context. A measure or indicator is valid if each of six criteria are met: (1) its construction is well-grounded in theory; (2) its performance is consistent with that theory; it is (3) precise, (4) dependable, and (5) accurate within specified performance standards; and (6) its accuracy is attributable to the well-grounded theory for that purpose and context”. These six concepts are generally attributed to: face or content validity (criterion no. 1), construct validity (criterion no. 2), test–retest reliability (criteria no. 3 and 4), and criterion validity (criteria no. 5 and 6) (2, 9). Therefore, the term validation is used to encompass various dimensions that must be assessed and considered to determine whether a given method is suitable for a specific purpose (2). This is particularly important when dealing with self-administered dietary assessment tools, which present greater challenges and complexities (2).

In the development and validation of dietary assessment tools, greater emphasis is often placed on their pragmatic and statistical validity, while other dimensions receive comparatively less consideration. However, as highlighted in the literature, a test “should not only be valid, but it should also appear valid” (10). The *appearance of validity* represents only one of the multiple interpretations associated with the term face validity, which has often generated confusion due to the variety of meanings attributed to it (11). To address this, Allen et al. recently proposed a working definition of face validity as “the clarity, relevance, difficulty, and sensitivity of a test to its intended audience” (11). This notion differs from content validity, defined as “the degree to which individual items capture the theoretical content domain of a construct” (11, 12). Evaluating whether an item is consistent with the construct definition (i.e., whether it adequately represents the intended content) is distinct from evaluating whether it is understandable, relevant, and appropriate for the intended audience. Both aspects should be assessed separately, as each plays an essential role in the construction and development of new tools (11).

To the best of our knowledge, these two dimensions are rarely incorporated into the validation process of dietary assessment tools. A recent systematic review by Cavero-Redondo et al. (13) identified 30 studies validating instruments to assess NOVA-defined UPF intake, including the study protocol of the present tool (8). While content validity was reported in most studies, none assessed face validity (13). Notably, within the Italian context, evidence on these aspects remains limited, with no studies explicitly addressing face validity and unclear reporting of content validity.

To help address these gaps, the present study aimed to assess face and content validity of the modified NOVA Food Frequency Questionnaire (mNOVA FFQ), a novel tool designed to assess UPF consumption and estimate total dietary intake of macro- and micronutrients in Italy, in order to refine it and establish a final version of the questionnaire to be subsequently evaluated for reliability and criterion validity.

## 2 Methods

### 2.1 Ethics and study design

This study constitutes the first phase of a multicenter project, promoted and conducted by the Italian National Institute of Health and the University of Udine. The project aims to develop and validate an FFQ specifically designed to estimate the UPF consumption and daily intake of macro- and micronutrients in the Italian adult population, starting from a critical reassessment of the NOVA classification and referring to previously validated tools (8). The project protocol, approved by Ethics Committee of the Italian National Institute of Health (approval n AOO 0040859 on 26 September 2024), has been presented in detail elsewhere (8).

**Figure 1:**
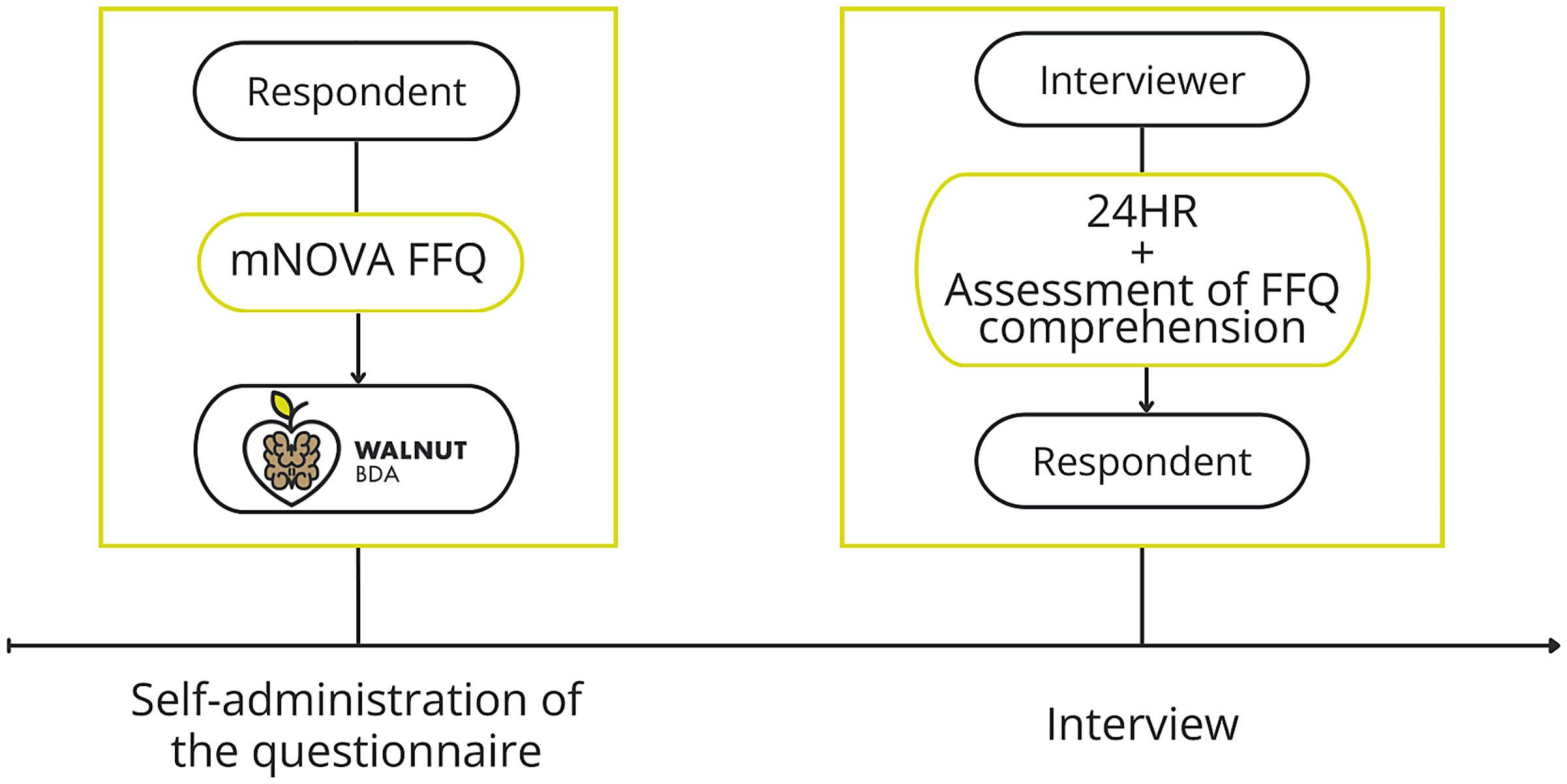
Design of the pilot study consisting of two consecutive phases: (1) Self-administration of the modified NOVA food frequency questionnaire (mNOVA FFQ) on the web-application Walnut BDA; (2) Online or in-person semi-structured interview. 24HR, 24 h dietary recall.

The present pilot study consists of two consecutive phases: (1) self-administration of a preliminary version of the mNOVA FFQ, and (2) online or in-person semi-structured interview (Figure 1).

### 2.2 Participants

A pilot study represents a small-scale preliminary investigation carried out prior to the actual research study, designed to address many aspects of the latter (14, 15). Since it is not primarily aimed at testing hypotheses, no formal sample size calculation is required; consequently, pilot studies are typically conducted on relatively small samples (14, 15). Although many studies consider a sample size of around 30 participants sufficient for similar pilot studies (14–16), the present study involved a convenience sample of 52 participants, providing a broader basis for preliminary evaluation. The final version of the questionnaire resulting from this first phase will subsequently be tested or reliability and criterion validity on an estimated sample of at least 436 subjects, as detailed in the protocol (8). Enrolment was conducted in selected workplaces (Italian National Institute of Health and University of Udine), between March and May 2025, including both employees and students of the institutions. Participants were volunteers aged ≥18 years, residing in Italy and with Italian citizenship. As the questionnaire is intended for the general population, individuals with a nutritional background (e.g., nutrition experts) were excluded.

### 2.3 Questionnaire

An initial version of the mNOVA FFQ to be used in this study, was developed based on tools previously validated in the Italian population and available in the literature (17–20). The questionnaire was developed following a critical reassessment of the NOVA classification system, which led to the definition of mNOVA, a modified version in which groups 3 (Processed Foods, PFs) and 4 (Ultra-Processed Foods, UPFs) are each further divided into two subgroups (3a and 3b, 4a and 4b), based on the salt, sugar, and fat thresholds recommended by the Food Standards Agency (FSA) (21), as described in detail elsewhere (8). The development process of the questionnaire involved compiling a comprehensive food list derived from previously validated tools, differentiating items according to the mNOVA groups, and grouping similar foods into single items. Questions were then defined alongside response options for frequency, portion size, and product type. The resulting version was tested and refined through this pilot study (8).

The questionnaire was semi-quantitative, designed to assess food habits (i.e., food intake over the past year), and self-administered on the ‘Walnut-BDA’ web application (8, 22). It included three sections: socio-demographic data, anamnestic information, and dietary data. The first two sections each contained nine questions on demographics, socio-economic status, lifestyle, and recent clinical data. The final section included items from the new mNOVA FFQ (8).

The FFQ itself was organized into 8 parts, corresponding to 8 food and beverage groups: (A) beverages; (B) milk and dairy products; (C) cereals; (D) meat, fish, and eggs; (E) vegetables, legumes, and fruit; (F) oils, fats, and seasonings; (G) sweets; and (H) other (8). It included 67 main branching questions, each addressing a food or beverage item and requiring a yes/no response on its consumption. In the case of an affirmative answer, follow-up questions were automatically triggered to collect information on portion size (choosing between small, medium, and large portions, according to national quantitative standards for portions (23)), frequency of consumption (choosing from 10 different options, ranging from ‘never’ to ‘>5 times per day’), and type of product usually consumed (e.g., brand). Moreover, each main item was further disaggregated into more specific sub-items reflecting distinct food subtypes within the same category (e.g., the main item ‘biscuits’ was broken down into four sub-items: homemade/artisanal refined flour biscuits, packaged refined flour biscuits, homemade/artisanal wholegrain biscuits, and packaged wholegrain biscuits), resulting in a total of 116 food items on which usual consumption information was collected. All questions were mandatory, except those asking about the brands of the most consumed products. The number of main questions and food items has been slightly expanded compared to the study protocol (8), in order to adapt the FFQ to the web application structure.

Selected participants were contacted by e-mail with a request to complete the questionnaire, after the acceptance of informed consent.

### 2.4 Interview

After completing the questionnaire, a qualified interviewer contacted participants to schedule an online or in-person interview. The semi-structured interview consisted of two parts: (1) the administration of 12 questions (11 in a yes/no format and one open question on completion time), designed to assess respondents’ understanding and interpretability of the mNOVA FFQ (e.g., clarity of instructions, options for frequency and portion size, descriptions of food items) and to evaluate its face validity using a qualitative method (11, 24); (2) the administration of a 24 h dietary recall (24HR), aimed at better understand the eating habits of the FFQ target population, specifically regarding UPF consumption, and informing potential adjustments to the FFQ’s content and closed list of food items (8). During the first part of the interview, participants were asked to justify their answers, which were simultaneously recorded on an *ad hoc* developed Microsoft Excel form by the interviewer. Another dedicated form was developed to record the information collected through the 24HR, a retrospective and short-term dietary assessment method referring to intake during the 24 h preceding the interview (25).

### 2.5 Analysis

In this study, quantitative and qualitative analyses were conducted to assess the content and face validity of the questionnaire.

Answers of the main yes/no questions of the FFQ were analyzed in terms of absolute frequency and percentage (%). For items with a negative response rate of over 50%, a comparison was made with data obtained from 24HRs and with food consumption data from the Italian national dietary survey on adult population (IV SCAI ADULT) (26, 27), to adjust the closed list of food items if necessary.

Data obtained from both sections of the interview were analyzed separately. The initial part of the interviews was analyzed thematically using the Braun and Clarke’s six-phase thematic analysis framework (28, 29), which includes (1) familiarization with data, (2) generating initial codes, (3) searching for themes, (4) reviewing themes, (5) defining and naming themes, and (6) writing the report. This allowed to summarize the feedback from the participants, in order to evaluate face validity and define the improvements to be made accordingly. The yes/no answers to the interview questions were also reported as absolute frequencies and percentages (%) for categorical variables, and as means with standard deviations (SD) for continuous variables. The 24HRs were analyzed using the Food Composition Database for Epidemiological Studies in Italy (BDA) (30), estimating the daily intake of total energy (kcal/day) and macronutrients (g/day). Results were reported as medians with interquartile ranges (IQR; Q1–Q3). The foods recorded for each participant were classified according to the mNOVA classification (8). The daily intake for each category was calculated, in terms of absolute intake quantity (g/day and kcal/day) and proportion of total energy intake (%).

In order to analyze dietary data from the FFQ and 24HR, food composition data for new items to be added to the BDA were calculated, using the BDA compilation approach (30). This process started from nutritional labels collected from the websites of major Italian retailers, recipe definition and, where possible, composition data from other databases.

All analyses were performed using R Core Team (2025) software 4.5.1 and Microsoft Excel (Microsoft 365, 2025).

## 3 Results

A total of 52 participants were enrolled in the present study, with an equal number of females and males (*n* = 26 each). The majority of the sample were 20 to 39 years old (59.6%) and reported a high educational level (88.5%). Table 1 shows the sociodemographic characteristics of the sample.

**Table 1:** Sociodemographic characteristics of the participants in the pilot study aimed at assessing the content and face validity of a food frequency questionnaire (*N* = 52).

Sample characteristics	Total (*N* = 52)	Females (*n* = 26)	Males (*n* = 26)
Age groups			
20–29 years	13 (25.0)	8 (30.8)	5 (19.2)
30–39 years	18 (34.6)	8 (30.8)	10 (38.5)
40–49 years	10 (19.2)	5 (19.2)	5 (19.2)
50–59 years	8 (15.4)	4 (15.4)	4 (15.4)
≥60 years	3 (5.8)	1 (3.8)	2 (7.7)
Education			
High school diploma	6 (11.5)	2 (7.7)	4 (15.4)
Bachelor’s degree	4 (7.7)	2 (7.7)	2 (7.7)
Master’s degree	25 (48.1)	14 (53.8)	11 (42.3)
Postgraduate qualification	17 (32.7)	8 (30.8)	9 (34.6)
Occupation			
Master’s students, research fellows, PhD students, medical residents	7 (13.5)	5 (19.2)	2 (7.7)
Administrative and managament support occupations	8 (15.4)	7 (26.9)	1 (3.8)
Professional, scientific, and highly specialised occupations	24 (46.2)	7 (26.9)	17 (65.4)
Technicians, officials	7 (13.5)	4 (15.4)	3 (11.5)
Other	6 (11.5)	3 (11.5)	3 (11.5)
Annual income*			
EUR 6,000 - 12,000	1 (2.2)	1 (4.8)	0
EUR 12,000 - 18,000	2 (4.3)	1 (4.8)	1 (4)
EUR 18,000 - 24,000	11 (23.9)	5 (23.8)	6 (24)
EUR 24,000 - 36,000	19 (41.3)	8 (38.1)	11 (44)
>EUR 36,000	13 (28.3)	6 (28.6)	7 (28)
*Missing*	6	5	1
Family size			
1 person	11 (23.9)	5 (19.2)	6 (23.1)
2 people	14 (26.9)	6 (23.1)	8 (30.8)
3 people	15 (28.8)	7 (26.9)	8 (30.8)
4 people	9 (17.3)	6 (23.1)	3 (11.5)
5 or more people	3 (5.8)	2 (7.7)	1 (3.8)
Macro-area of birth			
Northwest	1 (1.9)	0	1 (3.8)
Northeast	23 (44.2)	11 (42.3)	12 (46.2)
Centre	17 (32.7)	9 (34.6)	8 (30.8)
South	6 (11.5)	3 (11.5)	3 (11.5)
Islands	3 (5.8)	2 (7.7)	1 (3.8)
Foreign country	2 (3.8)	1 (3.8)	1 (3.8)
Macro-area of residence			
Northwest	1 (1.9)	0	1 (3.8)
Northeast	25 (48.1)	13 (50)	12 (46.2)
Centre	25 (48.1)	12 (46.2)	13 (50)
South	1 (1.9)	1 (3.8)	0
Islands	0	0	0

*Percentages are calculated on the total of subjects excluding missing data.

### 3.1 Content validity

**Figure 2:**
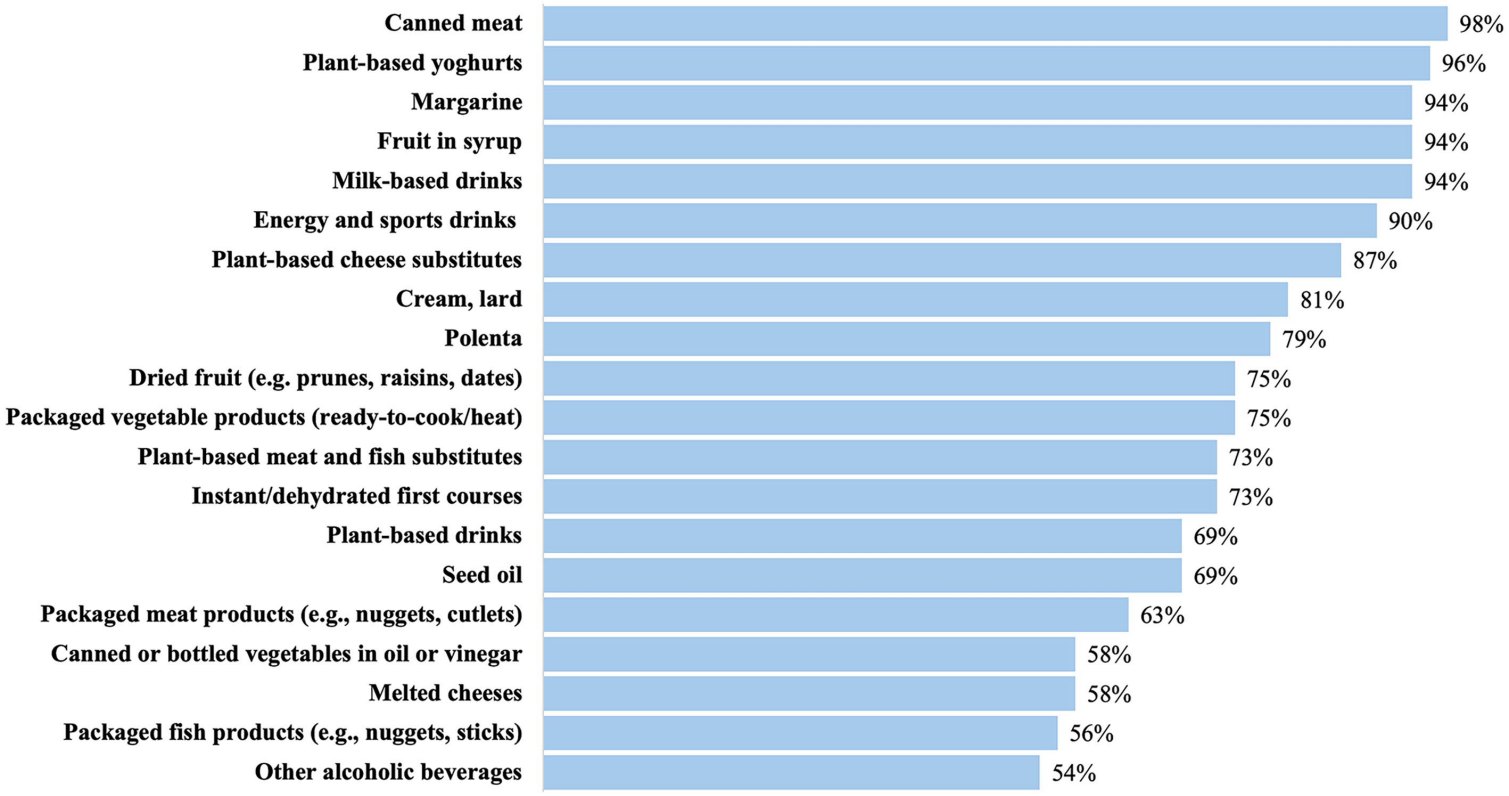
Items with predominantly negative responses (>50%) on food and beverage consumption main questions in the mNOVA FFQ. The remaining percentage of responses were positive indicating a habitual consumption (*N* = 52).

In order to identify the items to be retained, eliminated or modified, the questionnaire responses were analyzed. The absolute and percentage frequencies of negative responses to the 67 main questions on food and beverage consumption given by respondents are reported in Supplementary Table 1. A total of 20 items exhibited predominantly negative responses (>50%) to the main questions (Figure 2). Notably, canned meat emerged as the least consumed item among respondents, with a negative response rate of 98%.

Based on the analysis of the 24HRs, a comprehensive list of foods and beverages consumed by the participants was compiled and subsequently compared with the items included in the questionnaire (Table 2). All items reported in the 24HRs were represented in the FFQ, indicating a comprehensive coverage of the participants’ diet. Thirteen FFQ items were not consumed by participants in the 24 h preceding the interview; of these only one (i.e., sauces) did not correspond to the least consumed items listed in the questionnaire.

**Table 2:** Correspondence between main food frequency questionnaire (FFQ) items and foods and beverages recorded in 24 h dietary recalls (24HRs).

Food and beverage groups	Main FFQ items	Foods recorded in 24HRs
A. Beverages		
1	Coffee	**✓**
2	Tea and other hot beverages	**✓**
3	Wine	**✓**
4	Beer	**✓**
5	Other alcoholic beverages	**✓**
6	Soft drinks	**✓**
7	Energy and sports drinks	**✗**
B. Milk and dairy products		
8	Milk	**✓**
9	Milk-based drinks	**✗**
10	Yoghurt	**✓**
11	Fresh cheeses	**✓**
12	Aged cheeses	**✓**
13	Melted cheeses	**✓**
C. Cereals and cereal products		
14	Breakfast cereals	**✓**
15	Bread	**✓**
16	Bread substitutes	**✓**
17	Dried pasta and grains	**✓**
18	Fresh pasta	**✓**
19	Gnocchi	**✓**
20	Stuffed pasta	**✓**
21	Lasagne	**✓**
22	Instant/dehydrated first courses	**✗**
23	Polenta	**✗**
24	Potatoes and other tubers	**✓**
25	French fries, potato croquettes and similar	**✓**
26	Pizza	**✓**
D. Meat, fish, eggs		
27	Red meat	**✓**
28	White meat	**✓**
29	Processed meat (e.g., smoked, dried, cured meat)	**✓**
30	Sausages, wurst, and similar	**✓**
31	Canned meat	**✗**
32	Packaged meat products (e.g., nuggets, cutlets)	**✗**
33	Fresh or frozen fish, crustaceans, and shellfish (plain/natural)	**✓**
34	Preserved fish, crustaceans, and shellfish (e.g., smoked, dried, brined, canned)	**✓**
35	Packaged fish products (e.g., nuggets, sticks)	**✗**
36	Egg	**✓**
E. Vegetables, legumes, fruit		
37	Fresh or frozen vegetables (plain/natural)	**✓**
38	Salad (e.g., leaf lettuce)	**✓**
39	Canned or bottled vegetables (e.g., tomato sauce)	**✓**
40	Canned or bottled vegetables in oil or vinegar	✓
41	Packaged vegetable products (ready-to-cook/heat)	✓
42	Legumes (fresh, dried, canned/bottled)	**✓**
43	Fresh fruit	**✓**
44	Fruit in syrup	**✗**
45	Dried fruit (e.g., prunes, raisins, dates)	**✓**
46	Fruit jams	**✓**
47	Fruit juices and drinks	**✓**
48	Nuts and seeds	**✓**
F. Oils, fats, condiments		
49	Olive oil	**✓**
50	Seed oil	**✓**
51	Butter	**✓**
52	Margarine	**✗**
53	Cream, lard	**✓**
54	Sauces (e.g., ketchup, mayonnaise, béchamel)	**✗**
55	Sauces and condiments (e.g., pesto, meat sauce, tomato sauce)	**✓**
G. Sweets		
56	Biscuits	**✓**
57	Brioche, croissants, snacks	**✓**
58	Cakes and spoon desserts	**✓**
59	Ice cream	**✓**
60	Confectionery	**✓**
61	Sugar and other sweeteners	**✓**
H. Other		
62	Plant-based drinks	**✓**
63	Plant-based yoghurts	**✗**
64	Plant-based meat and fish substitutes	**✗**
65	Plant-based cheese substitutes	**✗**
66	Packaged savoury snack	**✓**
67	Fast food	**✓**

These data were then evaluated against national consumption data from IV SCAI survey (26, 27). This comparison revealed that, among the 13 items previously identified as minimally consumed, only four (i.e., canned meat, polenta, milk-based drinks, and energy and sports drinks) were actually consumed by less than 1% of the Italian adult population. Consequently, these four items were removed from the FFQ, while the remaining ones were retained in the questionnaire, preserved as individual items or aggregated with comparable food categories to optimize the questionnaire structure. For example, margarine, identified as an infrequently consumed fat source, was consolidated with butter to form a single composite main item.

The estimated daily intake of energy, macronutrients, fibre, and alcohol from the 24HRs is presented in Supplementary Table 2. In the total sample, median energy intake was 1798 kcal/day (IQR 1485–2,205), with carbohydrates, proteins, and fats contributing approximately 46.7, 16.0, and 36.5% of total energy, respectively. Supplementary Table 3 shows the consumption of foods and beverages, according to the mNOVA classification. PFs (group 3) accounted for 37.9% of total energy intake (IQR 23.4–55.0), with 13.2% (IQR 2.5–21.9) and 21.6% (IQR 8.0–38.6) corresponding to groups 3a (PFs with a nutritional profile below the FSA thresholds) and 3b (PFs with at least a nutrient above the FSA thresholds), respectively (see Supplementary Figure 1). UPFs (group 4) represented 20.4% of total energy intake (IQR 10.6–29.8), with the 6.8% (IQR 0–15.7) and 9.7% (IQR 3.1–20.5) corresponding to groups 4a and 4b, respectively (see Supplementary Figure 2).

### 3.2 Face validity

Frequencies of participants’ responses to the questions in the first part of the interview are presented in Table 3. All respondents considered the initial instructions clear and the section titles pertinent (questions 1 and 4). The mean completion time was 28 (±14) minutes and was deemed appropriate by 76.9% of the sample. Participants with shorter completion times indicated that they had omitted the optional items, responding primarily to the mandatory questions. Conversely, participants with longer completion times reported having completed both the mandatory and optional items. Most of the sample found the questions and response options easy to understand and interpret (questions 6–9) and considered the food descriptions and related examples to be appropriate (question 5). Fewer than half of the respondents had suggestions for improving the questionnaire (questions 10–12).

**Table 3:** Answers to interview questions to verify the understandability of the questionnaire (*N* = 52).

Interview questions		Answers	
		Yes	No
1	Do you find the initial instructions clear and suitable for understanding how to complete the questionnaire correctly?	52 (100.0)	0
2	How long did it take you to complete the questionnaire (in minutes)?*	28.0 (14.0)	
3	Do you find the time required to complete the questionnaire appropriate?	40 (76.9)	12 (23.1)
4	Do you find the names of the sections pertinent?	52 (100.0)	0
5	Do you find the descriptions of the foods and the related examples appropriate?	47 (90.4)	5 (9.6)
6	Did you easily identify the foods referred to in the questions?	51 (98.1)	1 (1.9)
7	Do you find the questions, in general, easy to understand and interpret?	51 (98.1)	1 (1.9)
8	Was it easy to understand and choose among the response options for the food consumption portion sizes?	45 (86.5)	7 (13.5)
9	Was it easy to understand and choose among the response options for the food consumption frequency?	43 (82.7)	9 (17.3)
10	Do you have any suggestions for improving the structure of the questionnaire (e.g., organization of sections and questions, grouping of foods, etc.)?	12 (23.1)	40 (76.9)
11	Do you have any suggestions for improving the content of the questionnaire (e.g., section names, food descriptions and examples, etc.)?	21 (40.4)	31 (59.6)
12	Do you have any suggestions for improving the general quality of the questionnaire?	15 (28.8)	37 (71.2)

Data are presented as absolute frequencies (*n*) and percentages (%).

*Data are presented as mean (SD).

A thematic analysis was undertaken of participants’ comments when assessing the face validity of the questionnaire. The analysis revealed eleven domains (Table 4): (1) completion instructions; (2) completion time; (3) section headings; (4) food description and examples; (5) clarity and recognizability of food items; (6) comprehensibility and interpretability of questions; (7) clarity and usability of portion size response options; (8) clarity and usability of consumption frequency response options; (9) structure of the questionnaire; (10) content clarity and adequacy; (11) overall quality of the questionnaire.

**Table 4:** Summary of qualitative feedback provided by the interview participants (*N* = 52).

Domain	Illustrative participant feedback (quotes)	Recommended actions
Completion instructions	1. *“While filling out the questionnaire, I forgot some of the instructions provided at the beginning.”* 2. *“The instructions did not suggest completing the questionnaire at home. If I had been at home, it would have been easier because I would have had the required information readily available.”*	1. Provide additional instructions within the different sections. 2. Suggest filling out the questionnaire at home.
Completion time	*“The questionnaire is relatively long and quite detailed, which make it time-consuming to complete.”*	Reduce the number of questions and food/drink items.
Section headings	*“During completion, I found myself anticipating certain foods, because I did not know whether they would be asked in later sections or what the subsequent items would be.”*	Provide an overwiew of the different section and items in the questionnaire.
Food description and examples	1. *“For items with few or no examples, I was somewhat unsure which food or drink they referred to.”* 2. *“For some foods, I noticed that the description was less detailed than for others”*	1. Include further food examples. 2. Harmonize the level of detail in the food descriptions for all items.
Clarity and recognizability of food items	1. *“I would have preferred to see more commercial product names, so that I could easily recognize the items.”* 2. *“Providing examples that clarified which food or drinks should be included or excluded within each item was particularly useful. It would be helpful to have more such examples.”*	1. Make reference to commercial product names, when relevant. 2. Provide additional examples specifying which foods or drinks should be included or excluded within each item.
Comprehensibility and interpretability of questions	*“Overall, the questions are clear, but some parts - like the health status section - are more challenging to interpret because of the language.”*	Use simpler language that is suitable for the general population.
Clarity and usability of portion size response options	*“The inclusion of household units was very helpful; however, adding pictures of serving sizes would further improve clarity.”*	Include pictures of serving sizes to facilitate identification.
Clarity and usability of consumption frequency response options	*“It has not always been easy to identify the right frequency of consumption as an annual average, especially for foods and drinks that are consumed only occasionally.”*	Provide further guidance on how to calculate consumption frequency, including for occasional consumption.
Structure of the questionnaire	*“I would have expected to find some items in other sections.”*	Consider reorganizing the items within the various sections.
Content clarity and adequacy	*“Questions regarding the brand name and type of product usually consumed were somewhat redundant and made the questionnaire heavier to complete.”*	Review and refine the questions concerning brand names and type of product usually consumed.
Overall quality of the questionnaire	*“I did not realize that it was possible to complete the questionnaire in multiple sessions.”*	Improve guidance on completing the questionnaire across multiple sessions to facilitate a more manageable experience for respondents.

Regarding the completion instructions, some participants reported forgetting the initial guidance during completion, and others noted that completing the questionnaire at home would have been easier, as relevant information would have been readily available. The completion time was perceived as appropriate, although the length and level of details made the questionnaire time-consuming. Concerning section headings, some respondents reported anticipating items that were in subsequent sections, as the structure did not always clearly indicate which foods would follow. Some participants commented on the structure of the questionnaire, noting that they would have expected certain items in other sections.

Feedback on the domain ‘food descriptions and examples’ indicated that items with few or no examples caused some uncertainty about which foods or drinks to include, and that some foods were described in greater detail than others. Similarly, in terms of clarity and recognizability of food items, participants suggested that including more commercial product names or additional examples would facilitate identification. Regarding comprehensibility and interpretability of questions, certain sections (e.g., health status) appeared to be particularly challenging for the general population to understand.

For portion size response options, participants suggested that images of serving sizes (i.e., small, medium, large) could improve clarity. The consumption frequency response options were sometimes challenging to use, particularly when estimating annual averages for foods that are occasionally consumed. Feedback on content clarity and adequacy highlighted that questions regarding brand names or types of products were sometimes redundant and increased the burden of completion. Finally, regarding the overall quality of the questionnaire, some participant reported being unaware that it was possible to complete the FFQ in multiple sessions.

As summarized in Table 4, several actions to improve the questionnaire were defined based on participants’ feedback.

## 4 Discussion

The main objective of the present study was to assess content and face validity of the mNOVA FFQ.

The most common methods to evaluate content validity typically rely on the recruitment of external and independent “subject matter experts” (31). In this study, however, content validity was established through logical and empirical analyses to verify the extent to which the questionnaire items represented the relevant content domain and supported the expected interpretations, in accordance with the Standards for Educational and Psychological Testing by the American Educational Research Association (32). Instead, face validity was assessed through a qualitative approach, using one-to-one interviews and a thematic analysis framework (11, 28, 29).

Specifically, findings from this pilot study enabled refinement of the closed list of food items and the questionnaire structure, while enhancing the clarity and understandability of the mNOVA FFQ, by expanding and harmonizing food descriptions and examples, simplifying language for a general population audience, adding section-specific instructions and guidance on frequency calculation, integrating visual aids for portion size identification, and revising questions on brand names and product types.

Comparing the data collected through the 24HRs with those from the FFQ, it has been observed that the questionnaire captured all foods and beverages consumed by participants, thereby supporting its comprehensive coverage. Analysis of the 24HR data largely confirmed the consumption patterns identified by the FFQ regarding infrequently consumed food items, with the notable exception of sauces, which were not recorded in the 24HRs but were not classified among the least-consumed items according to the FFQ data. This discrepancy likely reflects that a single day of dietary intake is not representative of individual consumption pattern, but this comparison was adequate for the purpose of the study (33).

The examination of the infrequently consumed items in this pilot study (*N* = 52) against the consumption patterns derived from the IV SCAI survey (26, 27), resulted in the identification of four foods and beverages. These four items were removed from the FFQ, while the remaining ones were retained in the questionnaire. Their removal is not expected to compromise the accuracy of UPF intake assessment, as they represent foods rarely consumed in the Italian population and the questionnaire is designed to reflect the dietary habits of this specific population. The decision to retain the remaining least-consumed items was primarily motivated by the fact that the majority of them are potentially UPFs. Among these, plant-based alternatives warrant particular attention, as their evaluation is essential for documenting the transition toward more sustainable plant-based dietary patterns, even when consumption levels are low (34).

Energy and nutrient intakes, as well as mNOVA group consumption, are reported solely to contextualize the overall diet of the FFQ target population. Notably, UPF intake in this study accounted for 20.4% of total energy, closely approximating the 23% observed in the latest national consumption data (35). However, considerable heterogeneity in consumption patterns across participants emerged from 24HRs, further underscoring the need for a specifically designed tool to capture more accurate intake estimates. This analysis also revealed the predominant consumption of subgroups 3b and 4b, representing processed and ultra-processed foods with an unbalanced nutritional profile, compared to FSA thresholds (21), respectively, as defined by the mNOVA classification (8). Consistent with previous literature (36, 37), this finding reinforces the necessity of further differentiation within the broad NOVA food categories (5) to better characterize dietary quality and health implications. It was this identified need that originally prompted the proposal of the simple and practical mNOVA classification in a prior study (8), and these results further underscore the demand for a novel and more structured food processing-based system that integrates nutritional aspects.

Although most participants considered the questionnaire clear and generally easy to understand, the interview feedback highlighted several areas where targeted improvements could be implemented. The recommended actions resulting from these comments represent concrete and pragmatic steps to enhance both usability and measurement quality. In particular, the revisions to completion instructions, section headings, and overall structure are expected to improve navigation through the questionnaire. The reduction in the number of items, together with clearer guidance on consumption frequency and the possibility to complete the questionnaire over multiple sessions, may further increase feasibility and reduce respondent burden, potentially limiting missing data. Importantly, many of these changes directly target sources of measurement error. The inclusion of additional examples and details in the descriptions of foods, the clarification of which foods should be included or excluded under each item, and the use of simpler language tailored to the general population are likely to reduce misclassification and improve content coverage. Similarly, the introduction of pictures of serving sizes and the refinement of questions about brand and product types address known difficulties in portion-size estimation and product recognition, which are key to obtaining valid intake estimates.

Overall, these findings underscore the importance of involving potential users of the tool in this methodological phase. Indeed, as evidence from previous studies similarly indicates, the target audience engagement is essential for developing valid and reliable tools that are clear and acceptable to those who will use them (24).

To the best of our knowledge, this is the first study specifically designed to assess the content and face validity of an FFQ designed to estimate UPF consumption. In light of the complexity and ambiguity surrounding the UPF concept, a further strength lies in the rigorous literature-based methodology adopted to develop a valid tool. Additionally, the collection of qualitative feedback from the target population through individual interviews represented a valuable source of information, providing practical insights into the usability of the instrument and informing targeted modifications to its structure, clarity, and applicability, thereby enhancing its overall future validity.

However, the study has several limitations that should be acknowledged. First, the small convenience sample introduced potential selection bias, as participants predominantly had a high educational level. To limit this bias and better reflect the intended target population, nutrition professionals were excluded at enrolment, given that the questionnaire is intended for use in the general population. Furthermore, the sample was balanced by sex, with equal representation of male and female participants. In a subsequent phase, the final version of the mNOVA FFQ will be evaluated in a larger and more heterogeneous sample, using a snowball sampling approach initiated through institutional mailing lists and social media platforms (8). Moreover, it is known that self-reported questionnaires may result in recall and misclassification biases, and the limited number of recall days per participant may not fully capture intra-individual variability in intake. To address misclassification biases, the questionnaire was refined based on the feedback received during this preliminary phase, including the addition of check questions to improve data accuracy (8). In addition, national consumption data were integrated to complement individual-level information and strengthen the robustness of intake estimates in the final version of the questionnaire. To mitigate recall biases, in this phase the 24HRs were administered by a trained interviewer, while in a subsequent validation phase, the questionnaire will be compared against a food diary, which represents the gold standard tool for real-time dietary recording (38). A further limitation is that content validity was not assessed through a formal quantitative approach involving the recruitment of external experts, as is usually done. Instead, it was established through logical and empirical analyses, grounded in the adaptation of previously validated questionnaires and the evaluation of item representativeness through comparison with 24HRs data and nationally representative food consumption data. Finally, no comparison between the FFQ and the 24HR was performed for energy and nutrient intakes, nor for the consumption of foods and beverages according to the mNOVA classification, as such analyses were not essential at this stage of the study. Indeed, the present work provides evidence only on content and face validity; further research is required to evaluate test–retest reliability and criterion validity of the final mNOVA FFQ version.

## 5 Conclusion

The present findings enabled the finalization of an FFQ designed to assess UPF consumption among Italian adults, through improvements in its content, structure, clarity and understandability.

This study underscores the importance of evaluating both content and face validity, as well as involving the target population in the development of new tools.

Further investigation is required to establish the reliability and criterion validity of the mNOVA FFQ for its application within the Italian adult population.

Once validated, this tool has the potential to serve as a valuable instrument in dietary surveys and epidemiological research, enabling a more nuanced characterization of food groups within the broad UPF category and advancing the understanding of their respective implications for health outcomes.

## Data Availability

The raw data supporting the conclusions of this article will be made available by the authors, without undue reservation.
