# Supplementary material for: Content and face validity of a food frequency questionnaire for the assessment of ultra-processed food consumption in the Italian adult population: a pilot study
Source: Front Nutr. 2026 Jun 5;13:1817542. doi: 10.3389/fnut.2026.1817542 (PMC13278918; doi:10.3389/fnut.2026.1817542)

Supplementary material

1 Supplementary Table 1 Negative answers to main questions in the Food Frequency Questionnaire relating to food and drink consumption. The remaining respondents answered positively.

Main FFQ items		Negative answers
**A. Beverages**		
1	Coffee	3 (5.8)
2	Tea and other hot beverages	10 (19.2)
3	Wine	15 (28.8)
4	Beer	21 (40.4)
5	Other alcoholic beverages	28 (53.8)
6	Soft drinks	20 (38.5)
7	Energy and sports drinks	47 (90.4)
**B. Milk and dairy products**		
8	Milk	19 (36.5)
9	Milk-based drinks	49 (94.2)
10	Yoghurt	11 (21.2)
11	Fresh cheeses	3 (5.8)
12	Aged cheeses	4 (7.7)
13	Melted cheeses	30 (57.7)
**C. Cereals and cereal products**		
14	Breakfast cereals	19 (36.5)
15	Bread	2 (3.8)
16	Bread substitutes	11 (21.2)
17	Dried pasta and grains	1 (1.9)
18	Fresh pasta	16 (30.8)
19	Gnocchi	17 (32.7)
20	Stuffed pasta	9 (17.3)
21	Lasagne	13 (25.0)
22	Instant/dehydrated first courses	38 (73.1)
23	Polenta	41 (78.8)
24	Potatoes and other tubers	2 (3.8)
25	French fries, potato croquettes and similar	21 (40.4)
26	Pizza	0
**D. Meat, fish, eggs**		
27	Red meat	5 (9.6)
28	White meat	6 (11.5)
29	Processed meat (e.g., smoked, dried, cured meat)	6 (11.5)
30	Sausages, wurst, and similar	22 (42.3)
31	Canned meat	51 (98.1)
32	Packaged meat products (e.g., nuggets, cutlets)	33 (63.5)

33	Fresh or frozen fish, crustaceans, and shellfish (plain/natural)	6 (11.5)
34	Preserved fish, crustaceans, and shellfish (e.g., smoked, dried, brined, canned)	11 (21.2)
35	Packaged fish products (e.g., nuggets, sticks)	29 (55.8)
36	Egg	4 (7.7)
**E. Vegetables, legumes, fruit**		
37	Fresh or frozen vegetables (plain/natural)	1 (1.9)
38	Salad (e.g., leaf lettuce)	7 (13.5)
39	Canned or bottled vegetables (e.g., tomato sauce)	15 (28.8)
40	Canned or bottled vegetables in oil or vinegar	30 (57.7)
41	Packaged vegetable products (ready-to-cook/heat)	39 (75.0)
42	Legumes (fresh, dried, canned/bottled)	5 (9.6)
43	Fresh fruit	0
44	Fruit in syrup	49 (94.2)
45	Dried fruit (e.g. prunes, raisins, dates)	39 (75.0)
46	Fruit jams	21 (40.4)
47	Fruit juices and drinks	17 (32.7)
48	Nuts and seeds	5 (9.6)
**F. Oils, fats, condiments**		
49	Olive oil	1 (1.9)
50	Seed oil	36 (69.2)
51	Butter	17 (32.7)
52	Margarine	49 (94.2)
53	Cream, lard	42 (80.8)
54	Sauces (e.g., ketchup, mayonnaise, béchamel)	17 (32.7)
55	Sauces and condiments (e.g. pesto, meat sauce, tomato sauce)	12 (23.1)
**G. Sweets**		
56	Biscuits	5 (9.6)
57	Brioche, croissants, snacks	19 (36.5)
58	Cakes and spoon desserts	16 (30.8)
59	Ice cream	3 (5.8)
60	Confectionery	7 (13.5)
61	Sugar and other sweeteners	12 (23.1)
**H. Other**		
62	Plant-based drinks	36 (69.2)
63	Plant-based yoghurts	50 (96.2)
64	Plant-based meat and fish substitutes	38 (73.1)
65	Plant-based cheese substitutes	45 (86.5)
66	Packaged savoury snack	14 (26.9)
67	Fast Food	17 (32.7)

Data are presented as absolute frequencies and percentages n(%).

Supplementary Table 2 Energy, macronutrients and alcohol intake: results from 24 h dietary recalls.

	**Total sample (n=52)**	**Males (n=26)**	**Females (n=26)**
**Energy (kcal/day)**	1798 (1485- 2205)	1975 (1803 -2419)	1533 (1317 - 1776)
**Total protein (g/day)**	71.9 (60.1 - 90.8)	87.0 (67.8 - 100.8)	63.8 (57.2 - 76.2)
**Total fat (g/day)**	72.8 (58.6 - 88.9)	76.4 (63.1 - 96.7)	65.3 (52.3 - 80.2)
**Available carbohydrates (g/day)**	209.9 (142.9 - 273.0)	267.1 (213.5 - 313.7)	144.3 (112.8 - 206.4)
**Soluble carbohydrates (g/day)**	79.3 (56.5 - 108.1)	101.2 (71.7 - 127.1)	64.3 (49.0 - 82.1)
**Dietary total fibre (g/day)**	19.5 (15.9 - 23.8)	22.9 (19.0 - 28.1)	17.3 (15.8 - 19.6)
**Alcohol (g/day)**	0 (0 - 3)	1.2 (0 - 12.3)	0 (0 - 0)

Data are shown as median and interquartile range (Q1–Q3).

Supplementary Table 3 Food consumption of the participants categorized according to the mNOVA groups: absolute intake quantity (g/day and kcal/day) and proportion of total energy intake (%). Results from 24 h dietary recalls.

**mNOVA groups**		**Food consumption**		
		**g/day**	**kcal/day**	**% of total energy intake**
1		751.0 (613.3 - 1003.8)	480.0 (300.9 - 627.0)	24.8 (16.4 - 42.0)
2		18.5 (9.8 - 33.3)	153.3 (80.9 - 228.7)	8.0 (4.0 - 14.3)
3	3a	203.5 (57.5 - 307.5)	203.7 (45.1 - 456.9)	13.2 (2.5 - 21.9)
	3b	132.0 (50.0 - 227.3)	386.0 (146.4 - 765.6)	21.6 (8.0 - 38.6)
	3a+3b	305.0 (160.0 - 553.6)	698.1 (327.6 - 1121.9)	37.9 (23.4 - 55.0)
4	4a	43.8 (0 - 201.3)	117.9 (0 - 233.6)	6.8 (0 - 15.7)
	4b	41.5 (11.9 - 102.0)	189.0 (65.4 - 397.7)	9.7 (3.1 - 20.5)
	4a+4b	150.0 (51.8 - 283.8)	366.9 (186.0 - 550.9)	20.4 (10.6 - 29.8)

Data are shown as median and interquartile range (Q1–Q3).

4 **Supplementary Figure 1** Processed food group (group 3) intake, according to mNOVA classification. Proportion of total energy intake (%) is shown as median and interquartile range (Q1–Q3).

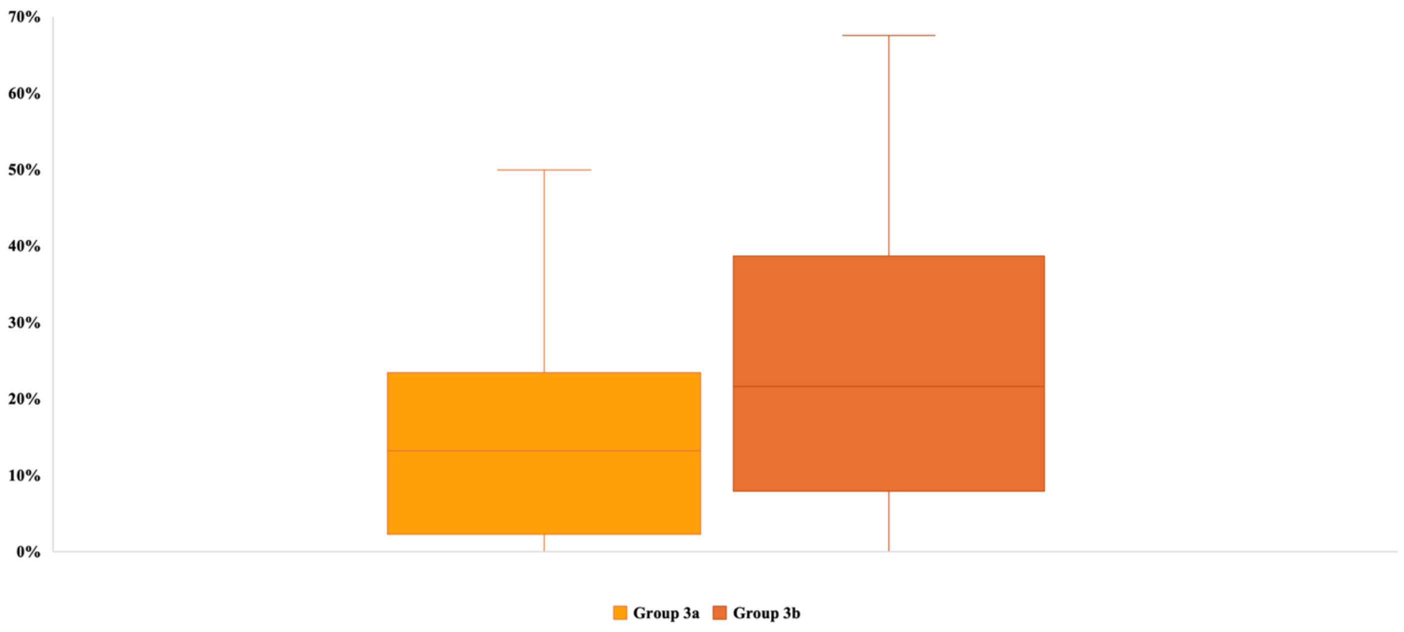

Supplementary Figure 2 Ultra-processed food group (group 4) intake, according to mNOVA classification. Proportion of total energy intake (%) is shown as median and interquartile range (Q1–Q3).

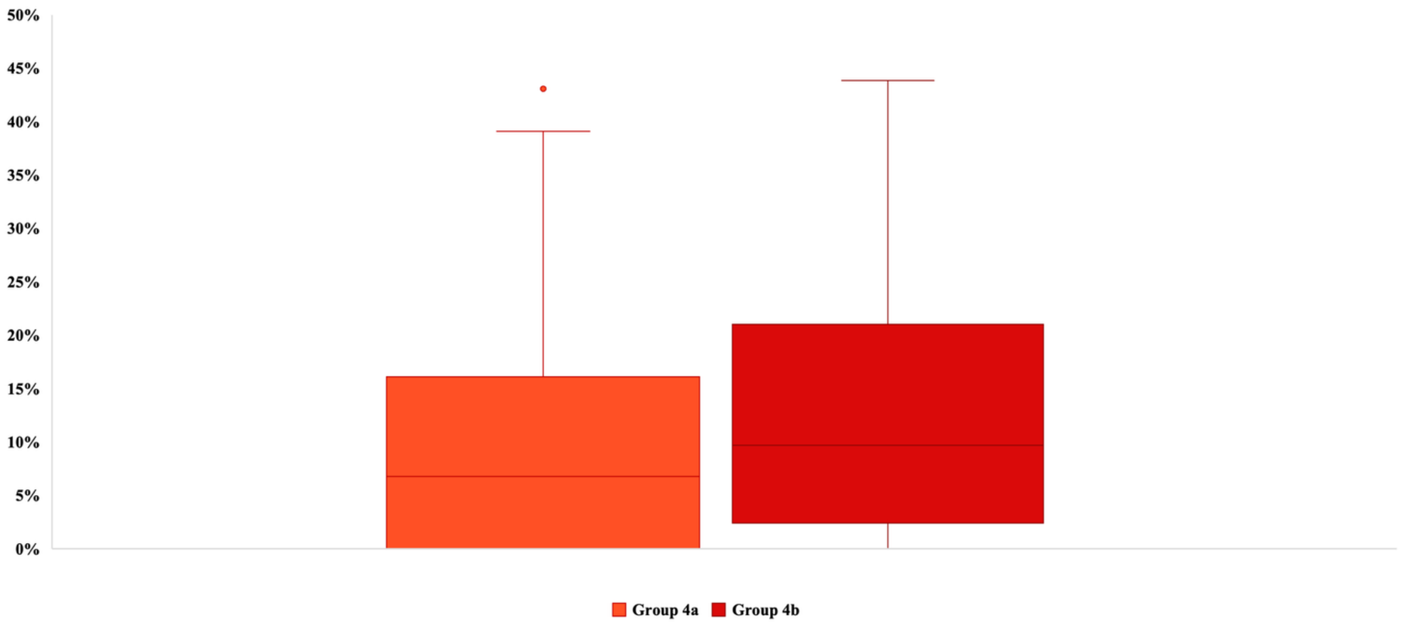

Supplement: Supplementary file 1 [file Data_Sheet_1.PDF]
